# Intubation of the Larynx

**Published:** 1887-10

**Authors:** Joseph O’Dwyer

**Affiliations:** New York


### *INTUBATION OF THE LARYNX.*

By Dr. JOSEPH O'DWYER, of New York.

He began experiments in 1880, at the New York Foundling Asylum. At that time, tracheotomy was in disfavor at the asylum, because for some time its usefulness had not been demonstrated by a single recovery. The difficulty and danger of tracheotomy deter many physicians from the operation, and not a few even of the laity fail to understand how a child's suffering can be relieved by cutting its throat.

He first used a prostatic catheter introduced by the nostril. It was found impracticable, partly because the patient would remove it with the hands. The long tube suggested a short one, and its practicability was almost at once demonstrated by the construction of a tube, one end of which extended into the trachea, while the other occupied the vestibule of the larynx, permitting the epiglottis to close over it in the act of swallowing. But what was to hold it down in place against the expulsive effort of a cough? First a bivalve form was given to its lower extremity, permitting of its expansion after introduction. But the tube was thus made a trap for fragments of pseudo-membrane. Longer tubes reaching nearly to the bifurcation of the trachea were kept in place better, but, after a cough, the long tube would be found thrown partially out of place, and had to be pushed back with the finger. A second shoulder was then added to the tube, below the expansion or head at its upper extremity. This shoulder kept the tube from being repelled, but the abruptness of its upper border made extraction very difficult.

The abrupt shoulder then gave way to a gradually tapering enlargement, which was found to accomplish all that could be desired. A tube constructed in this way, when projected upward by coughing, will slip back into position by the pressure of the vocal bands on the sloping sides, aided by its weight. This retaining swell is only made thick enough to hold the tubes loosely in the larynx, in order to permit of their easy expulsion in case of sudden occlusion by masses of pseudo-membrane too large to pass through. Owing partly to this and partly to the difference in the size of the larynx in different children of the same age, they will sometimes be expelled even when unoccupied by false membrane, and before the laryngeal stenosis is fully relieved. If it were not for this, such a tube could be indefinitely worn. A larger and shorter tube would not be expelled, but it would make serious pressure on the lining membrane of the larynx. Such a tube may be temporarily applied with advantage as a laryngeal dilator, not in ordinary severe croup, but in slow cases, where little help is required.

Intubation is apparently, but not really, a simple operation. With more than the usual dexterity and coolness, and an easy case, it will be called by the physician who tries for the first time, a very simple thing. With less dexterity and a difficult case to manage, it will be called a difficult operation.

When established, and perfected, and in common use, intubation can never be considered a satisfactory remedy, in view of the complications and the very nature of membranous croup. The first results, if good, will create enthusiam; if bad, distrust.

In comparing tracheotomy and intubation, the question is not which will save most life in a given number of cases submitted to treatment, but which operation can be performed or will be permitted in the greater number of cases.

Dr. F. E. Waxham, of Chicago, Ill., while strongly advocating intubation, would not overlook its disadvantages. The operation is a difficult one in its performance. The soft tissues may be wounded, or the trachea perforated. It is more difficult to extract than to insert the tube. A serious trouble may ensue from the difficulty of swallowing when the tube is in place. These are the dangers. None are so grave that they cannot be overcome.

On the other hand, it has many advantages over tracheotomy. It can be done almost instantly. There is no loss of blood, no pain, no shock, no open wound with the possibility of septicæmia or erysipelas. There is no drying of mucus in the tube, and no necessity for cleansing the tube. Less attention is required in the after-treatment. Finally, we can save as large a number of adults as, and a much larger number of children than, by tracheotomy.

After presenting a series of statistics, the writer closed with the statement that intubation has been performed 1,000 times within two years, and that 269 lives have been saved from certain death.

Dr. Charles G. Jennings, of Detroit, Mich., expressed high appreciation of the labors of Dr. O'Dwyer and Dr. Waxham, and believed this method to be of great value. His own experience, however, had led him to prefer tracheotomy. His thirty-six tracheotomies had been followed by seventeen recoveries, while he had applied the tube in twelve cases without a recovery. Dr. Waxham's statistics contain about 1,000 cases, with twenty-six per cent. of recoveries. He believed that certain operators in both operations have records far above the average, and that the successful tracheotomists have a larger portion of recoveries than the physicians performing intubation who rank as the most successful. He believed that the personal equation is an important element in considering the comparative value of the two procedures.

Although intubation possesses the advantages alleged in the papers read before the section, it remains true that tracheotomy has advantages which should not be forgotten. After an incision, the trachea is accessible for the removal of membrane and dried mucus, and direct medication by instillations and powers. Adequate nourishment after intubation is almost impossible, while there is no such difficulty after tracheotomy.

An obvious advantage of intubation is that it can be performed by many physicians who would not undertake a tracheotomy, and in this way relief, impossible otherwise, will be afforded in a great many cases. His own experience, however, leads him to urge tracheotomy in all cases offering reasonable chance of success, and to reserve intubation for the cases in which operative interference will give nothing more than euthanasia, and for those patients whose parents refuse consent to tracheotomy.

Dr. T. J. Pitner, of Jacksonville, Ill., questioned the correctness of Dr. Waxham's closing statement that the 249 cases of recovery following intubation were so many lives saved by the operation. This assumes that, that all cases of stenosis from croup or diphtheria are, without operative procedures, necessarily fatal. He did not, however, question the utility and great importance of this operation, which appears to him to be preferable to tracheotomy.

Dr. Northrup stated, in answer to questions, that in thirty-three cases he had never met the slightest difficulty in inserting the tube, and had not failed in a single case to fully and promptly relieve the dyspnœa. He had met with no accident, and probably gentlemen present had in the aggregate used the tube several hundred times and never met with untoward accidents. He thought the only real drawback to the success of intubation lay in its interference with feeding. It is sometimes difficult to keep up full nutrition when swallowing is so impeded.

In one of his cases, the physician in charge had retired hopeless, without doing tracheotomy, and being ignorant of intubation. The child was exhausted and comatose. The tube was inserted. The child returned to consciousness. At the end of forty hours, he coughed out the tube. It was not re-applied, and the child made a perfect recovery.

Dr. O'Dwyer, in answer to questions, said that, in his opinion, statistics will be of very little value in settling the question between tracheotomy and intubation, until a very large number have been obtained. Aside from the question of saving life, intubation will be resorted to in the most hopeless cases, those in which tracheotomy would not be thought of, for the sake of securing euthanasia.

